# Tuberculous pleural effusion in a patient with sympathetic ophthalmia on immunosuppression: a case report

**DOI:** 10.1186/s40794-021-00153-3

**Published:** 2021-10-15

**Authors:** Dharshana Thiagarajan, Daphne Ai Lin Teh, Nor Azita Ahmad Tarmidzi, Hamisah Ishak, Zamzurina Abu Bakar, Mae-Lynn Catherine Bastion

**Affiliations:** 1 Department of Ophthalmology, Hospital Kuala Lumpur, Ministry of Health Malaysia, Kuala Lumpur, Malaysia; 2 Department of Ophthalmology, Faculty of Medicine, Hospital Canselor Tuanku Muhriz, Kuala Lumpur, Malaysia; 3 Institute of Respiratory Medicine, Hospital Kuala Lumpur, Ministry of Health Malaysia, Kuala Lumpur, Malaysia

**Keywords:** Tuberculous pleural effusion (TPE), Tuberculosis, Immunosuppression, Pleural tuberculosis, Exudative

## Abstract

**Background:**

Tuberculous pleural effusion (TPE) is paucibacillary, making its diagnosis difficult based on laboratory investigations alone. We present a case of a patient with a TPE who was initially misdiagnosed to have azathioprine-induced lung injury. The diagnosis of TPE was arrived at with the help of clinical assessment, laboratory and radiological investigations.

**Case presentation:**

A 25-year-old chronic smoker with sympathetic ophthalmia on long-term immunosuppression, latent tuberculosis infection and a significant family history of tuberculosis presented with a three-week history of productive cough, low-grade fever, night sweats and weight loss. Examination of the lungs showed reduced breath sounds at the right lower zone. Chest x-ray showed minimal right pleural effusion with a small area of right upper lobe consolidation. The pleural fluid was exudative with predominant mononuclear leukocytes. Direct smears of sputum and pleural fluid; polymerase chain reaction of pleural fluid; and sputum, pleural fluid and blood cultures were negative for *M. tuberculosis* (MTB) and other organisms. As he did not respond to a course of broad-spectrum antibiotics, he was then treated as a case of azathioprine-induced lung injury. However, his condition did not improve despite the cessation of azathioprine. A contrast-enhanced computed tomography of the thorax showed right upper lobe consolidation with tree-in-bud changes, bilateral lung atelectasis, subpleural nodule, mild right pleural effusion and mediastinal lymphadenopathy. Bronchoalveolar lavage was negative for malignant cells and microorganisms including, MTB. However, no pleural biopsy was done. He was empirically treated with anti-tubercular therapy for 9 months duration and showed complete recovery.

**Conclusion:**

A high index of suspicion for TPE is required in individuals with immunosuppression living in regions endemic to tuberculosis. Targeted investigations and sound clinical judgement allow early diagnosis and prompt treatment initiation to prevent morbidity and mortality.

## Introduction

Sympathetic ophthalmia is a rare chronic bilateral diffuse granulomatous intraocular inflammation following penetrating injury or surgery to one eye. Systemic immunosuppression is the mainstay of treatment, with corticosteroids being the first-line. When steroid resistance or intolerable side effects occurs, second-line treatment with steroid-sparing agents can be administered [1–3]. However, the downside to systemic immunosuppression is the increased risk of the development of opportunistic infections such as tuberculosis, in addition to drug-related adverse events [4].

Tuberculous pleural effusion (TPE) is the second commonest form of extrapulmonary tuberculosis (EPTB) after tuberculous lymphadenitis [5, 6]. It is the commonest cause of exudative pleural effusion in countries endemic to tuberculosis, such as Malaysia [7, 8]. Patients with TPE typically present with acute or subacute symptoms of fever, unilateral pleuritic chest pain, cough, night sweats, dyspnoea and weight loss [9]. Immunosuppressive drug use, human immunodeficiency virus (HIV) infection, cirrhosis, malignancy, alcoholism, history of pulmonary tuberculosis (PTB), intravenous drug usage and chronic lung disease have been identified as risk factors for EPTB. The paucibacillary nature of tuberculous pleural fluid makes it a diagnostic challenge [10]. Hence, a high index of suspicion is required for early diagnosis and prompt treatment initiation in regions endemic to tuberculosis.

We now present a case of a patient presenting with sympathetic ophthalmia following penetrating ocular injury who developed a TPE during treatment.

## Case presentation

**Fig. 1:**
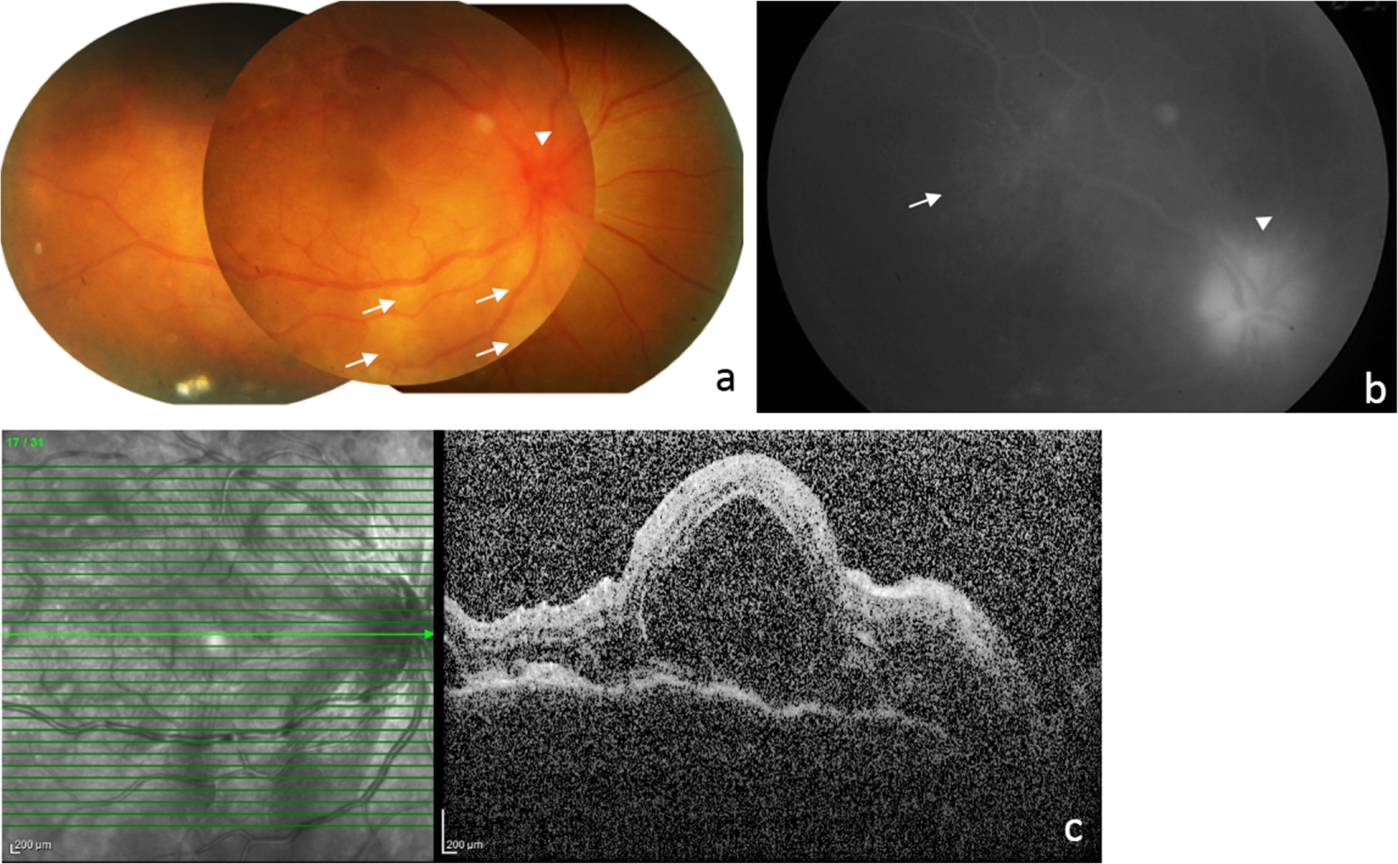
Fundus photograph of OD during the initial presentation (**a**) shows hazy media with the presence of vitritis, swollen and hyperemic optic disc (arrowhead), multifocal yellow-white choroidal lesions (arrows) and exudative retinal detachment involving the macula. Fluorescein angiography OD (**b**) shows multiple pinpoint hyperfluorescent lesions at the posterior pole and mid-peripheral retina (arrow) with a hyperfluorescent disc (arrowhead) in the late phase. Optical coherence tomography of the macula (**c**) shows multifocal serous detachment

A 25-year-old chronic smoker with no known co-morbidities presented in May 2019 with a 10-day history of right eye (OD) redness, discomfort, photophobia and reduced vision. He had a history of penetrating ocular injury with uveal prolapse to the left eye (OS) following a road traffic accident 4 years before presentation, for which primary repair was performed. He gave a history of occasional headaches but denied vomiting, hearing disturbances, skin changes, hair loss, chronic cough, hemoptysis, dyspnoea, night sweats or constitutional symptoms. His late father had passed away 5 years prior due to abdominal tuberculosis. His younger sister had a history of pulmonary tuberculosis 2 years prior. On examination, visual acuity was counting fingers OD and no light perception OS. The anterior segment examination of OD revealed an injected conjunctiva, non-granulomatous keratic precipitates (KP), plasmoid aqueous with cells of 4+ and seclusio pupillae with iris pigments on the lens. Limited fundus view of OD through the small, poorly dilating pupil showed vitritis of 1+, a swollen and hyperemic optic disc, multifocal yellow-white choroidal lesions at the posterior pole and mid-peripheral retina and multifocal exudative retinal detachment involving the macula (Fig. 1a).

The OS was phthisical, and the anterior segment details could not be appreciated. Intraocular pressure (IOP) was 14 mmHg OD and unrecordable OS. Fluorescein angiography (FA) showed multiple pinpoint hyperfluorescent lesions at the posterior pole and mid-peripheral retina with a hyperfluorescent disc in the late phase (Fig. 1b). Optical coherence tomography (OCT) of the macula showed multifocal serous detachment (Fig. 1c). Given the suggestive history and clinical findings, he was diagnosed with sympathetic ophthalmia. Other than a positive tuberculin skin test (TST) of 15 mm, other microbiological and radiological investigations were negative for tuberculosis and syphilis. Discussion with internal medicine counterparts concluded no contraindication for the initiation of corticosteroids despite him having latent tuberculosis infection (LTBI) as there was no recent contact with active tuberculosis cases, and corticosteroid use conferred only a low risk of tuberculosis activation. He was then started on topical dexamethasone 0.1% every 2 hours, topical atropine 1% TDS, and intravenous methylprednisolone (1 g/day for 3 days) along with a proton pump inhibitor, followed by oral prednisolone 50 mg daily (1 mg/kg/day).

**Fig. 2:**
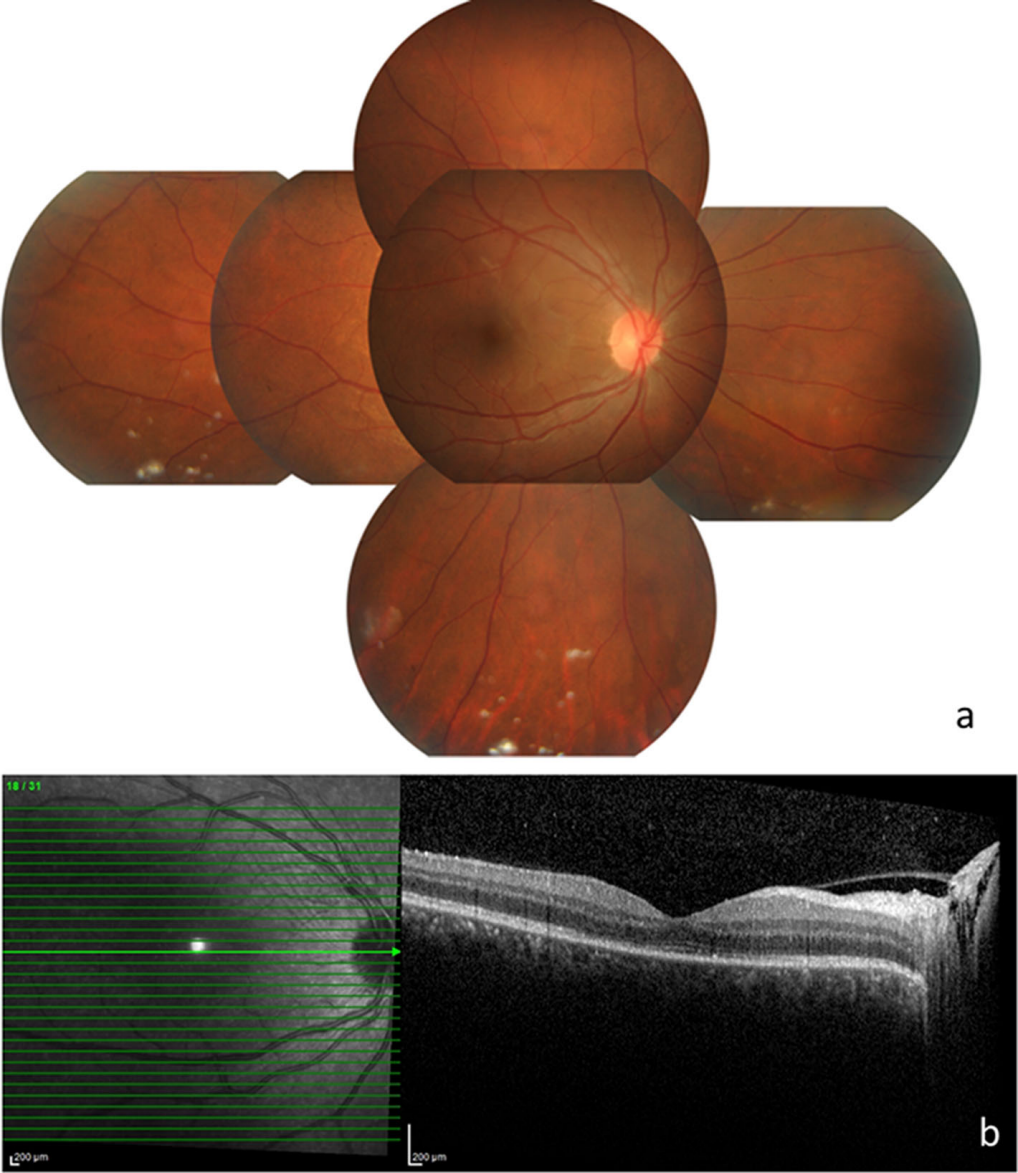
Fundus photograph of OD one month after initiating oral prednisolone (**a**) shows resolution of vitritis, choroidal lesions, exudative retinal detachment and optic disc swelling. Optical coherence tomography of the macula (**b**) shows resolved multifocal serous detachment

A month later, vision improved to 6/12 OD with improvement in anterior chamber reaction, and resolution of vitritis, choroidal lesions, exudative retinal detachment and optic disc swelling (Fig. 2). Oral azathioprine 50 mg BD (2 mg/kg/day) was commenced, while topical and oral steroids were slowly tapered and maintained at a low dose of oral prednisolone 10 mg and topical dexamethasone 0.1% daily. During treatment, he developed raised IOP and cataract as a result of steroid usage and was started on topical timolol 0.5% BD and topical dorzolamide 2% BD.

**Fig. 3:**
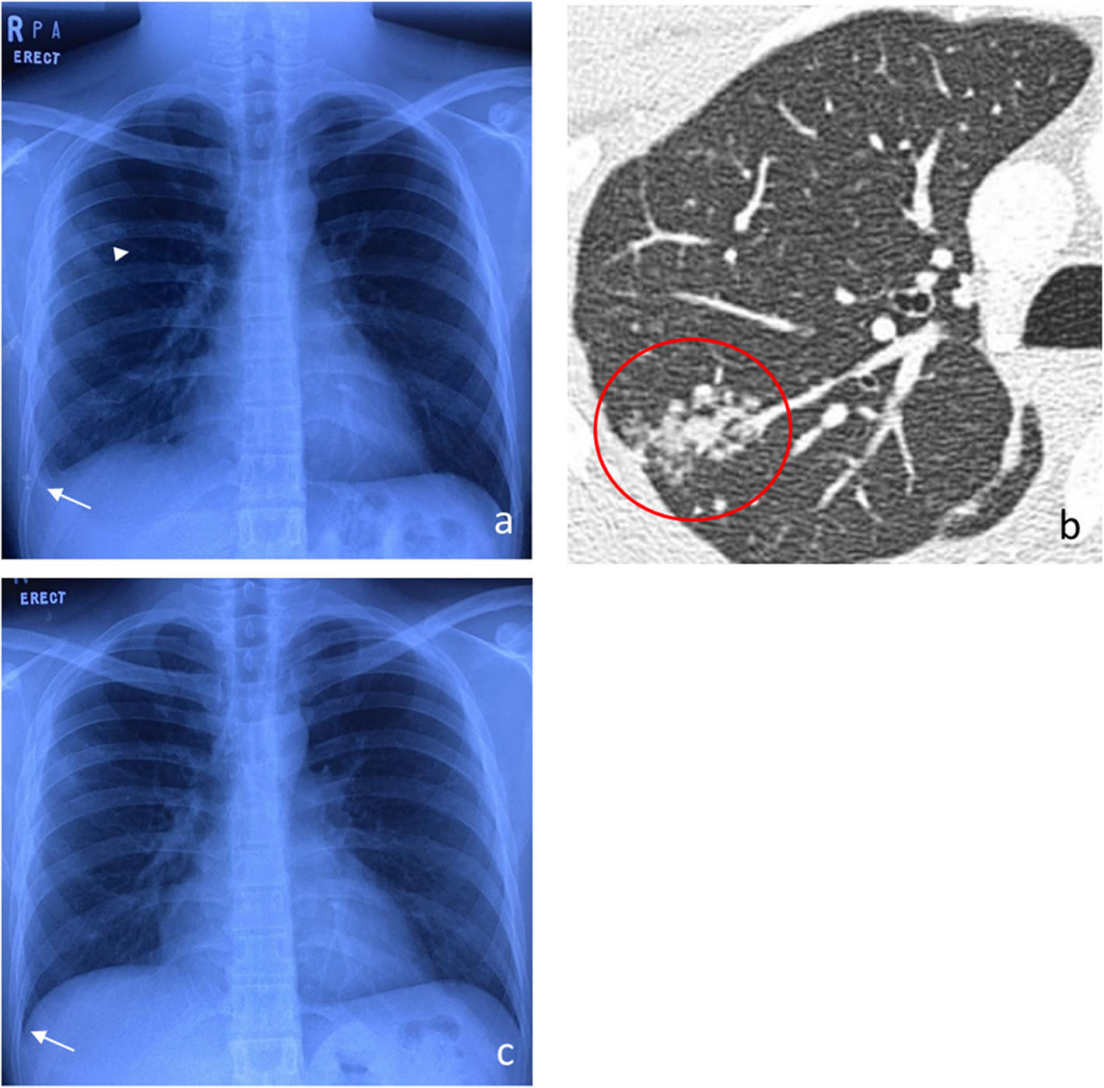
Initial chest X-ray (**a**) shows minimal right pleural effusion (arrow) with a small right upper lobe consolidation (arrowhead). Contrast-enhanced computerized tomography of the thorax (**b**) shows right upper lobe consolidation with surrounding tree-in-bud changes (circle). Repeated chest X-ray five months after ATT (**c**) shows the resolution of right lung consolidation and pleural effusion (arrow)

Seven months later, in December 2019, he presented with a three-week history of productive cough associated with intermittent low-grade fever, night sweats and weight loss of 5 kg. He did not have any ocular symptoms. Upon examination, he was comfortable, not tachypnoeic and not septic-looking. Breath sounds at the right lower zone of the lungs were reduced. There were no significant ocular findings. A chest X-ray showed minimal right pleural effusion with a small right upper lobe consolidation (Fig. 3a). Azathioprine was withheld due to the possibility of opportunistic infection, and he was admitted to the ward. 30 ml of straw-coloured pleural fluid was drained from a pleural tap. His blood investigations were as follows: white cell count 11.03 × 10^9^/L, haemoglobin 16.7 g/dL, platelet 477 × 10^9^/L, erythrocyte sedimentation rate (ESR) 21 mm/h, C-reactive protein 3.9 mg/L with normal liver and renal function tests. The pleural fluid was exudative (glucose 6.6 mmol/L, LDH 238 U/L, protein 59 g/L and pH 8) with few mononuclear leukocytes, no visible bacteria on Gram stain, and no *M. tuberculosis* (MTB) DNA detected on GeneXpert MTB/RIF assay. Both sputum and pleural fluid direct smears were negative for acid-fast bacilli (AFB), and cultures were negative for MTB at 7 weeks of incubation. Blood, sputum and pleural fluid bacterial cultures were negative as well. As he did not respond to a course of broad-spectrum antibiotics, he was treated as a possible case of azathioprine-induced lung injury pending further investigation.

A subsequent contrast-enhanced computed tomography (CECT) of the thorax a week later showed right upper lobe consolidation with surrounding tree-in-bud changes associated with patchy bilateral lung atelectasis, subpleural nodule, mild right pleural effusion and mediastinal lymphadenopathy (Fig. 3b). Bronchoalveolar lavage showed no AFB, fungal bodies, or malignant cells, and cultures were negative for bacteria and MTB. As the lung changes showed no improvement despite cessation of azathioprine for 5 months combined with the findings on CECT thorax, he was diagnosed with smear-negative pleural tuberculosis. He was treated with 9 months of anti-tubercular therapy (ATT) (2 months of isoniazid, rifampicin and pyrazinamide followed by 7 months of isoniazid and rifampicin). Subsequently, his symptoms markedly improved. Repeated chest x-ray 5 months after ATT initiation showed resolution of right lung consolidation and pleural effusion (Fig. 3c). Thirteen months after azathioprine was withheld, the right eye remained quiet. Visual acuity was 6/12 OD due to a posterior subcapsular cataract.

## Discussion

This case proved to be a diagnostic challenge. The diagnosis of sympathetic ophthalmia was reinforced by the positive response to immunosuppression alone without adjunctive antimicrobials or ATT. There were no episodes of reactivation throughout the 1 year that he was on immunosuppression alone. A differential diagnosis of ocular tuberculosis should be considered due to its endemicity in this region, its ability to present similarly, and the positive TST in this patient. However, ocular tuberculosis would have worsened with immunosuppression alone and without the appropriate administration of ATT [11]. This patient showed complete resolution of ocular findings without any recurrence.

Opportunistic infections are the commonest cause of lung changes in individuals with immunosuppression [12]. However, in the absence of a causative organism and no significant improvement with broad-spectrum antibiotic therapy, the differential diagnosis of drug-induced toxicity has to be considered. In our patient, the diagnosis of azathioprine-induced lung injury was entertained as all infective workup were negative, and he did not improve with a course of broad-spectrum antibiotics. He did not exhibit any of the common side-effects of azathioprine which include bone marrow suppression, nausea, vomiting, anorexia, hepatotoxicity and hypersensitivity reactions [13]. Azathioprine-induced lung injury is a rare dose-dependent adverse effect. It should be considered in patients on immunosuppression who present with fever, acute respiratory symptoms and hypoxia due to pneumonitis, and acute respiratory distress syndrome (ARDS) [12–14]. CT thorax in these patients shows nodular hyperdensities with ground-glass areas without pleural effusion or focal lung lesions [12, 14]. Clinical improvement usually occurs rapidly within 1 to 2 weeks of discontinuation of azathioprine [12–14]. In our case, the patient’s symptoms were insidious. He was afebrile, not hypoxic, and his CT thorax did not show any ground-glass appearance typically seen in azathioprine-induced toxicity. There was also no improvement despite azathioprine cessation. At this point, an alternate diagnosis was considered.

Due to his risk factors of immunosuppression, cigarette smoking and significant history of tuberculosis exposure, the possibility of pleural tuberculosis was considered. The insidious nature of his symptoms combined with the significant weight loss, CT thorax findings and presence of an exudative pleural effusion supported the diagnosis. However, the absence of MTB yield from any body fluid was a dilemma.

Our patient had a right-sided small pleural effusion. TPE are usually unilateral and can be of any size. A case series of 333 patients showed 86.5% having unilateral pleural effusions and 20.4% having small pleural effusions [15].

The CT thorax, in this case, revealed more findings compared to the initial chest radiograph. This is further supported by Zhai et al., who stated that CT thorax is more sensitive than chest radiography in detecting lung parenchymal changes in TPE, with approximately 40–85% of patients demonstrating parenchymal disease [15]. The CT thorax findings of pleural effusion, subpleural nodule, tree-in-bud changes, lung parenchymal consolidation and mediastinal lymphadenopathy can be found in tuberculosis [9, 16].

A predominantly neutrophilic TPE indicates an earlier MTB infection phase and a higher MTB yield than a predominantly lymphocytic TPE [17]. This would explain the presence of solely mononuclear leukocytes in our patient’s pleural fluid, as he already had the symptoms for a total of 3 weeks before sampling of the effusion. Unfortunately, it was not specified whether these mononuclear leukocytes were lymphocytes or monocytes. The absence of MTB yield in our patient is expected, given the longer illness duration.

TPE is paucibacillary, as it is a hypersensitivity reaction to mycobacterial proteins rather than frank infection [18]. This may also explain the lack of MTB detection from our patient’s pleural fluid analysis. Mycobacterial cultures from pleural fluid have a higher sensitivity for AFB (24–58%) than direct smears (< 5%) as direct smears would require a greater bacilli concentration of 10,000/mL compared to 10–100/mL required for culture [19, 20]. GeneXpert, a type of nucleic acid amplification test (NAAT), utilizes a polymerase chain reaction (PCR) method to detect small amounts of MTB genetic material in samples [9]. It has a pooled sensitivity of 50.9% against culture from pleural fluid [21]. However, pleural biopsy has the highest sensitivity in diagnosing pleural tuberculosis with either the presence of granulomas or detection of MTB from direct smear, cultures or NAAT (46–88%) [7, 8, 10, 19, 22]. Although a pleural biopsy may have provided a positive result, the positive clinical and radiological response to ATT, demonstrated in this case, is sufficient to make the diagnosis of pleural tuberculosis [22].

The decision to treat LTBI with ATT is multifactorial depending on the TB burden and economic status of the country, and risk stratification of an individual for tuberculosis activation [23]. According to the World Health Organization’s recommendation for screening and treatment of LTBI, corticosteroid administration and cigarette smoking alone confer a low risk for tuberculosis activation and there is no conclusive evidence of benefit from ATT prophylaxis in these groups of patients [23, 24]. However, the addition of azathioprine may increase this risk and hence, treatment of LTBI in an individual with multiple risk factors should be considered. Additionally, vigilance for reactivation of tuberculosis is required in patients with LTBI on immunosuppression.

## Conclusion

An infective cause should always be ruled out in an immunosuppressed patient presenting with respiratory manifestations. In regions endemic to tuberculosis, a high index of suspicion is required to diagnose TPE. Targeted investigations are essential to increase diagnostic yield. However, the absence of MTB from sampling should not hinder the diagnosis in the presence of suggestive clinical and radiological findings.

## Data Availability

Not applicable.

## Abbreviations

AFB: Acid-fast bacilli
ATT: Anti-tubercular therapy
BD: Twice a day
CECT: Contrast-enhanced computed tomography
ESR: Erythrocyte sedimentation rate
EPTB: Extrapulmonary tuberculosis
FA: Fluorescein angiography
HIV: Human immunodeficiency virus
IOP: Intraocular pressure
KP: Keratic precipitate
LTBI: Latent tuberculosis infection
MTB: *M. tuberculosis*
NAAT: Nucleic acid amplification test
OCT: Optical coherence tomography
OD: Right eye
OS: Left eye
PCR: Polymerase chain reaction
TDS: Thrice a day
TPE: Tuberculous pleural effusion
TST: Tuberculin skin test
